# The Use of a Virtual Reality Platform for the Assessment of the Memory Decline and the Hippocampal Neural Injury in Subjects with Mild Cognitive Impairment: The Validity of Smart Aging Serious Game (SASG)

**DOI:** 10.3390/jcm9051355

**Published:** 2020-05-06

**Authors:** Monia Cabinio, Federica Rossetto, Sara Isernia, Francesca Lea Saibene, Monica Di Cesare, Francesca Borgnis, Stefania Pazzi, Tommaso Migliazza, Margherita Alberoni, Valeria Blasi, Francesca Baglio

**Affiliations:** 1 IRCCS Fondazione Don Carlo Gnocchi ONLUS, via Capecelatro 66, 20148 Milan, Italy; mcabinio@dongnocchi.it (M.C.); frossetto@dongnocchi.it (F.R.); sisernia@dongnocchi.it (S.I.); fsaibene@dongnocchi.it (F.L.S.); mdicesare@dongnocchi.it (M.D.C.); fborgnis@dongnocchi.it (F.B.); malberoni@dongnocchi.it (M.A.); fbaglio@dongnocchi.it (F.B.); 2 Consorzio di Bioingegneria e Informatica medica (CBIM), 27100 Pavia, Italy; s.pazzi@cbim.it (S.P.); t.migliazza@cbim.it (T.M.)

**Keywords:** virtual reality, serious game, mild cognitive impairment, dementia, Alzheimer disease, digital biomarker, hippocampus, MRI, cognitive rehabilitation, computerized assessment

## Abstract

Due to the lack of pharmacological treatment for dementia, timely detection of subjects at risk can be of seminal importance for preemptive rehabilitation interventions. The aim of the study was to determine the usability of the smart aging serious game (SASG), a virtual reality platform, in assessing the cognitive profile of an amnestic mild cognitive impairment (aMCI) population, its validity in discriminating aMCI from healthy controls (HC), and in detecting hippocampal degeneration, a biomarker of clinical progression towards dementia. Thirty-six aMCI and 107 HC subjects were recruited and administered the SASG together with a neuropsychological evaluation. All aMCI and 30 HC subjects performed also an MRI for hippocampal volume measurement. Results showed good usability of the SASG despite the low familiarity with technology in both groups. ROC curve analyses showed similar discriminating abilities for SASG and gold standard tests, and a greater discrimination ability compared to non-specific neuropsychological tests. Finally, linear regression analysis revealed that the SASG outperformed the Montreal cognitive assessment test (MoCA) in the ability to detect neuronal degeneration in the hippocampus on the right side. These data show that SASG is an ecological task, that can be considered a digital biomarker providing objective and clinically meaningful data about the cognitive profile of aMCI subjects.

## 1. Introduction

Virtual reality (VR) has been defined as an application that allows users to navigate and interact with a three-dimensional computer-generated environment in real time [1]. Paralleling the advancements of information technology (IT) in the implementation of multi-dimensional platforms for the care of patients [2,3,4], VR gives the opportunity to improve cognitive assessment allowing more ecological and smart instruments of evaluation [5,6,7,8,9,10]. In particular, serious games (SGs), defined as “digital applications specialized for purposes other than entertaining” [11,12], with their capacity to implement VR environments can represent an easily-accessible method to assess cognitive functions in a more ecological way, since they can host complex environments resembling real-life context with different levels of complexity [7,9,10,13].

Although the field of SGs is quite young, digital applications for clinical purposes are becoming more and more available, and some of them have been validly used in healthy subjects [10,14,15] as well as in clinical populations, particularly in Parkinson’s disease [9,16] and in subjects with amnestic mild cognitive impairment (aMCI) and dementia [17,18,19,20,21,22]. Data from the literature confirm that SGs are not only appropriate but also recommended for the assessment and stimulation of elderly people with MCI and dementia [23].

Faced with the increasing number of new evaluation instruments, the need to implement scientifically valid, reliable and smart instruments to detect clinical and pre-clinical conditions in the early stages is of pivotal importance.

MCI is a “mild neurocognitive disorder” [24,25] lying on the continuum between normal aging and cognitive decline [26,27], affecting approximately 10% to 20% of adults over 65 years of age [26]. MCI is defined as a symptomatic pre-dementia stage in which the cognitive impairment does not affect the functional activities of daily living [26]. The incidence for the development of dementia in individuals with MCI older than 65 years of age is approximately 4.9% in two years [28]. Among the different forms of MCI, the amnestic form (aMCI) refers to a condition in which the memory dysfunction predominates [29] and that is associated with specific brain changes, such as reduction of hippocampal volume [30,31] and cortical thinning in medial temporal [30] and parietal [32,33] cortices. These abnormalities are specific for aMCI and represent biomarker of neuronal degeneration [34]. In particular, the reduction of the hippocampal volume is currently considered a biomarker to detect the subjects with aMCI at higher risk of cognitive decline [35,36]. Consequently, with their brain changes aMCI subjects have a higher risk of developing Alzheimer’s disease (AD), when compared to non-amnestic MCI [37]. This strong association with dementia makes aMCI an important target for early pharmacological and rehabilitation interventions such as cognitive and physical exercise training [38,39]. In this line, at the beginning of 2018 the American Academy of Neurology published the new practice guidelines, underlying the importance to assess people in this pre-clinical condition using validated tools, detecting both functional impairment and cognitive status in a longitudinal way [28]. For large scale and timely screening for aMCI, more ecological tools, mimicking everyday activities, are becoming a cardinal issue. In this line, an ecological, virtual 3D environment-based tool named “smart aging” (smart aging serious game, SASG-http://www.cbim.it/en_new/serious-games-en/index.html [7,10]), aimed at evaluating multiple cognitive domains, was developed. The SASG integrates five cognitive tasks in a setting resembling a real house. Subjects are asked to play the tasks of the game following simple instructions while moving through the rooms, while the software evaluates performance accuracy and reaction times. When tested in a large cohort of aged cognitively-preserved subjects, SASG was demonstrated to be a valid tool for assessing cognitive functions [10]. The interface of SASG is specifically designed to be easily accessible to older or non-expert computer users by means of a touchscreen, a first-person perspective, and an automatic navigation system.

According to these premises, the present study had a three-fold aim: 1. to evaluate the usability of SASG in a cohort of aMCI patients and healthy controls (HC), taking into account the familiarity with the used technology; 2. to investigate the validity of SASG in discriminating between aMCI and healthy control subjects in comparison with gold standard pencil paper neuropsychological tests; and 3. to determine the validity of the SASG in detecting hippocampal degeneration as a neuroimaging marker of neuronal injury in comparison to gold standard pencil paper neuropsychological tests.

## 2. Materials and Methods

### 2.1. Recruited Sample

A total sample of 139 subjects participated in the study. Subjects with a diagnosis of aMCI (*n* = 32) were consecutively recruited from the outpatient memory clinic at the IRCCS Fondazione Don Carlo Gnocchi ONLUS (FDG, Milan, Italy). The inclusion criteria were: (1) aMCI diagnosis according to the recommendations of the National Institute on Aging [27] and the DSM 5 diagnostic criteria [24]; (2) presence of a mini-mental state examination [40] score ≥ 24, corrected for age and years of education according to Italian normative data [41]; (3) age ≥ 65 years and school attendance ≥3 years; (4) abnormal memory function confirmed by an informant and documented by the neuropsychological examination; (5) no impairment in functional activities of daily living as determined by a clinical interview with both the patient and the caregiver; (6) absence of psychiatric illnesses, with particular attention to depressive symptoms (Hamilton depression rating scale score ≤ 12 [42]) and severe behavioral disturbance; (7) absence of severe auditory/visual loss that can prevent from the use of technological device and from the execution of the serious game; (8) absence of major brain abnormalities at MRI scan or significant cerebral vascular diseases (Hachinski score above 4) [43].

A sample of age-, gender- and education-matched HC (*n* = 107) was also included. HC were obtained from the CBIM repository and from volunteers recruited from FDG. In more detail, HC were recruited from universities of the third age, social clubs and among volunteers working in the FDG and caregivers of outpatients. All the HC lived independently, had active social and cognitive lives and were native Italian speakers. They underwent an in-clinic neuropsychological evaluation including MMSE score and a neurological interview to exclude major neurological complaint. They fit the inclusion criteria number 3, 5, 6 and 7 of the above listing and presented a MMSE ≥ 28. The study was approved by the Ethics Committee of the Don Gnocchi Foundation and all subjects signed a written and informed consent.

### 2.2. Neuropsychological Evaluation

All subjects performed in a clinical setting and in close proximity with SASG completion, a neuropsychological evaluation. This was conducted by a trained neuropsychologist using conventional pencil–paper test including:

[i] the Montreal cognitive assessment test (MoCA): an established rapid cognitive screening tool able to differentiate MCI from normal aging and from AD patients, with a high sensitivity and specificity [44,45]. Raw data were corrected according to Italian normative data [46].

[ii] the immediate and delayed recall scores obtained from the free and cued selective reminding test (FCSRT, [47]), a widely used memory test that provides details on the encoding and retrieval phases of the memorization processes. Data were corrected according to [48].

[iii] the trail making test (TMT, [49]), versions A and B, for the assessment of executive functions and mental flexibility, as well as visual search, and processing speed. Data were corrected according to [50].

### 2.3. Serious Game Task: The Smart Aging Serious Game (SASG)

Each subject was asked to complete, in a clinical setting, a single SASG (http://www.cbim.it/en_new/serious-games-en/index.html) session, extensively described elsewhere [6,7,10]. Shortly, the SASG was administered in presence of a neuropsychologist and was performed using a touch-screen monitor, in a first-person perspective. SAGS is an ecological serious game based on a virtual house, in which subjects are asked to interact with the different parts of the scenario and to perform specific tasks. All the actions performed by the subjects within the SAGS are recorded and measured, allowing the assessment of memory, executive functions, working memory, and visual spatial processes [10] through the execution of five tasks. Task 1 (T1), named “Object search”, investigates memory, spatial orientation and attention; task 2 (T2), called “Water the flowers while listening to the radio”, assesses executive functions and divided attention; task 3 (T3), “Make a phone call”, evaluates executive functions, selective attention, working and perspective memory; task 4 (T4), “Choose the right object”, investigates memory and task 5 (T5), “Find the objects”, assesses long-term memory (recall), spatial orientation and attention.

In order to familiarize with the virtual environment and the use of the touch screen before the actual evaluative session, subjects naïve in the use of ICT and touch screens, were presented with a 10-min interactive demo. Successively, no other feedback was provided while the subjects were performing the serious games.

In line with Bottiroli (2017) [10], we collected accuracy (accuracy index, AI) and time (time index, TI) measurements for each SAGS task. AI and TI were then converted into z-scores considering the mean and standard deviation of the HC sample. For each task a total score was computed (as the difference between AI and TI, in line with [10] and the sum of the total scores of all tasks was computed to calculate the Smart Aging Total Score (SASG-Total).

Computer familiarity measures were collected with an ad hoc questionnaire according to [51]. Specifically, each subject was asked to fill out a computer questionnaire concerning its familiarity with computers and touch-screen use, expressed in terms of frequency of use, before SASG session.

### 2.4. MRI Acquisition and Analysis

All of the aMCI performed a single brain MRI acquisition (1.5 T Siemens Magnetom Avanto, Erlangen, Germany) within two weeks from the neuropsychological evaluation, to collect a high-resolution 3D-T1 image (MPRAGE; TR/TE = 1900/3.37 ms, FoV = 192 mm × 256 mm, in-plane resolution 1 mm × 1 mm, slice thickness = 1 mm, number of axial slices = 176) in order to measure hippocampal volumes. The MRI protocol included a dual-echo turbo spin echo proton density PD/T2-weighted image (repetition time (TR) = 5550 ms, echo time (TE) = 23/103 ms, matrix size = 320 × 320 × 45, resolution 0.8 × 0.8 × 3 mm^3^) for the evaluation of the white matter hyperintensities.

High-resolution T1 images have been analyzed using Freesurfer’s recon-all pipeline (https://surfer.nmr.mgh.harvard.edu/, [52]) and total hippocampal volumes have been segmented using the hippocampal subfield segmentation tool of Freesurfer (v.6.0) [53], basing on a statistical postmortem atlas built primarily upon ultra-high resolution (~0.1 mm isotropic) MRI data. Quality checks were performed at each step of the pipeline, and at the end of the cortical parcellation according to ENIGMA guidelines (http://enigma.ini.usc.edu). Total intracranial volume (TIV) has been computed using Freesurfer automatic subcortical segmentation, on the basis of the probabilistic aseg atlas [54]. Hippocampal volumes were then normalized for the total intracranial volume obtaining a normalized value (n-Hipp), using a proportional approach [55].

### 2.5. Statistical Analyses

Statistical analyses have been performed using MedCalc 18.5 (http://www.medcalc.org). Descriptive statistics included relative and absolute frequencies for categorical variables, median and IQ range for non-normally distributed continuous measures and means and standard deviation (SD) for continuous measures. The normality of data distribution was assessed considering the skewness and kurtosis coefficients and an appropriate parametric/non-parametric test was used for statistical analyses. When appropriate, data were corrected for multiple comparison using the Bonferroni correction, dividing the α-value (0.05) by the number of statistical tests on dependent variables.

Direct comparisons (one-way ANOVA or Mann–Whitney) on age, gender and educational level were performed to assess the between-groups matching for these variables. In order to assess the presence of hippocampal degeneration, n-Hipp volume of aMCI group was compared to an internal dataset of 30 healthy subjects with comparable age, gender and education and with the same inclusion criteria defined in the Section 2.1.

For aim 1, i.e., evaluating the usability of SASG, a between-groups comparison was performed on the results of the computer and touch screen familiarity questionnaire with a chi-squared analysis. The key performance indicator considered for the usability of the platform was the percentage of subjects that completed the SASG evaluation (all 5 tasks). Moreover, in order to test the influence of the familiarity with computers and the SASG score, a 2 × 2 ANOVA with clinical group (aMCI vs HC) and frequency of computer use factor (infrequent vs. frequent) on SASG total score data was performed.

For aim 2, that is, determining the validity of SASG in discriminating between aMCI and HC, we performed between groups direct comparisons (one-way ANOVA or Mann–Whitney) for SASG, for the conventional pencil–paper test used to detect aMCI (MoCA total score, FCSRT scores), and for pencil paper–tests not focused on mnemonic functions (TMT-A and B). To further investigate the validity of SASG, a ROC (Receiver Operating Characteristics) curve analysis was performed to determine differences in the sensitivity and specificity of SASG in comparison with MoCA, FCSRT, TMT A and B. On the basis of our ROC curves, the best cut-off score for SASG in discriminating between HC and MCI was also investigated (Youden J index).

Finally, for aim 3, i.e., determining the ability of SASG to detect the hippocampal neuronal loss, a partial correlation analysis was performed between SASG Task total score (SASG-Total) and n-Hipp and between MoCA test and n-Hipp. Age, gender and years of education were included as a covariate of no interest. Only tasks that resulted significantly correlated with n-Hipp volume were entered into a linear regression analysis.

## 3. Results

### 3.1. Demographics and SASG Usability

Demographics of the recruited sample are detailed in Table 1. No between-group differences in age, education and gender were found. As expected, the two groups differed in the MMSE score. Moreover, aMCI subjects showed significantly lower n-Hipp volume bilaterally compared to the HC group belonging to the MRI internal database (*n* = 27; mean age 73.59 ± 4.88 years; nine males, mean education 11.59 ± 3.81 years; MMSE 29.33 ± 0.89). Due to movement artifacts, two subjects with aMCI were excluded from MRI data analyses (*n* = 30; mean age 76.07 ± 4.73 years, 15 males, mean education 10.87 ± 3.80; MMSE 27.69 ± 1.76).

Results from the computer familiarity scale (Table 2) shows no differences between groups in the frequency of computer use, with 53.12% of aMCI and 60.38% of HC subjects who never used a PC, while the remaining had a frequent use (at least weekly). The frequency of use of a touch screen was comparable between groups: 62.5% of aMCI and 72.65% of HC who had never used a touch screen before the participation in the study; 18.75% of aMCI and 20.75% of HC who used it unfrequently (not more than once a month); and 18.75% of aMCI and 6.60% of HC who had a frequent (at least weekly) use. All the subjects from both groups completed the five tasks of the SASG session indicating an appropriate level of usability of the digital tool also for a population of aMCI. Testing the influence of familiarity on SASG score our ANOVA 2 × 2 results shows significant group effect (F(1134) = 64.109, *p* < 0.001), however, the effect of frequency of use factor was not significant (F(1134) = 2.975, *p* = 0.087) and no significant interactions were found (F(1134) = 0.74, *p* = 0.391), indicating that familiarity with the use of the PC did not influence SASG score.

### 3.2. Neuropsychological Assessment Results

Data relative to the neuropsychological evaluation (Table 3) reveal that for the MoCA test total score the aMCI group performance was within the normal range (see cut-off values in Table 3) but significantly worse than the HC. As for the memory performances, assessed with the FCSRT test, the aMCI group performed worse than controls and below the cut-off value in all four indices assessing immediate and delayed free and total recall memory. On the contrary, the performances at the TMT A and B test were in the normal range and comparable between groups.

### 3.3. SASG Results

Results of the SASG (Table 4) show significant differences between groups in the accuracy of all SASG tasks, with the exception of T2 and T3, and in the time indices of all subtests except the T4. SASG-total is significantly lower in the aMCI group.

**Figure 1:**
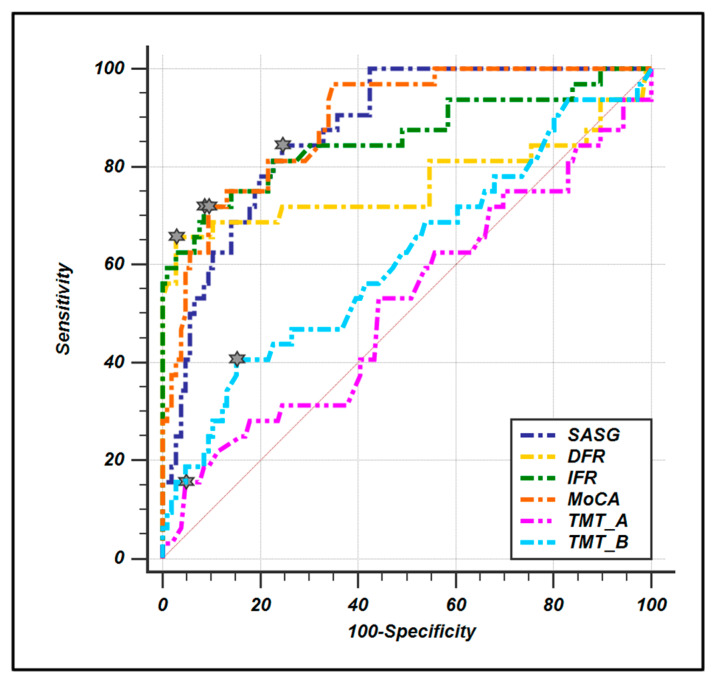
ROC curve comparison between smart aging total score and conventional paper-and-pencil test. Little stars indicate the criterion associated to Youden J statistic.

ROC curves were computed to evaluate the diagnostic sensitivity and specificity of SASG-total and all the pencil–paper neuropsychological tests (Figure 1, Table 5). The results show high values for both parameters for all tests except the TMT A and B. Moreover, the ROC comparison analysis reveals that SASG-total is comparable to MoCA and FCSRT in the ability to discriminate between groups, while the comparison with TMT A and B reveals significantly higher ability for the SASG-total.

### 3.4. SASG, MoCA and Hippocampal Volume

When investigating the presence of neuronal degeneration through n-Hipp volume, we found a significant volumetric reduction in aMCI compared to HC subjects bilaterally (Table 1). Moreover, results of the partial correlations between SASG-total and neuropsychological variables (MoCA, FCSRT and TMT) with hippocampal volume reveal significant correlation between right n-Hipp volume and the serious game. No significant relation is present with MoCA score FCSRT and TMT after Bonferroni correction (Table 6).

Finally, a linear regression analysis to evaluate the predictive value of SASG to determine hippocampal volume reveals a significant relationship between right n-Hipp and SASG-total (R^2^: 0.14; *p*-value: 0.042).

## 4. Discussion

The recent technological advancements in digital medicine have fostered the development of innovative tools for a better care of people’s health and wellbeing [56]. In the last years, several lines of research have led to the development of innovative ICT solutions to perform cognitive evaluation with the use of SGs, virtually reality based instruments able to reproduce more ecological environments, in both healthy and neurological populations [5,7,8,9,10,16,23]. Recent data demonstrated the usability and efficacy of SG for the early detection and monitoring of cognitive impairment in neurodegenerative disorders [57].

The first aim of the present study was to test the usability of an innovative virtual reality tool, the smart aging serious game (SASG) platform, in a cohort of aMCI subjects.

In our sample, the majority of subjects (whether aMCI or HC) had never used a PC and a touch screen before performing the SASG session. Despite this finding, no effects of familiarity were found on the SASG score and all the participants were able to go through the whole SASG session in a clinical setting, indicating a good level of usability. This result confirms the good usability of the platform for our sample. Indeed, SASG interface was specifically implemented for older and non-expert users and did not necessitate skilled abilities [6,10]. All the aspects of the usability of the platform were considered in a previous work [6] and several technical precautions were considered. For instance, to overcome the difficulties in navigating through 3D scenarios, the touch screen was found to be more usable than the mouse. Moreover, these data suggest the possibility to also use the SASG platform remotely from the patient’s home. This last use, though, will require a dedicated validation.

The second aim of the study was to determine the validity of the SASG in discriminating between a preclinical population with aMCI and successful aging subjects. Data on well-established pencil–paper tests confirmed the amnestic profile of the aMCI population that showed reduced scores in the MoCA test, and in the immediate and delayed FCSRT subtests and relative total scores [58]. On the contrary, as expected no differences between groups were found in the TMT tests, a task specifically targeted to measure visual–motor skills, mental flexibility, processing speed and sequencing [59]. This confirms the specificity of the amnestic impairment of the aMCI a preclinical condition with a high risk to develop AD type of dementia. The cognitive profile using SASG depicts a picture similar to the one observed with conventional paper-and-pencil tests. In detail, considering only the accuracy (i.e., independently from the time of execution), aMCI have a reduced performance in all tasks involving mnemonic functions (T1, T4 and T5) with preserved competences in tasks involving mainly executive functions and attention (T2 and T3). When considering the time of execution (i.e., independently from the accuracy), results show a slowdown in aMCI in all tasks except task 4, the only one presenting with a reduced visual complexity due to the bi-dimensional aspect of the scenario and thus not requesting an increased cognitive effort to deal with the greater graphical complexity of a 3D-environment [7,10]. Given the comparable unfamiliarity with technology in both groups, the reduced performances in the aMCI versus HC subjects can be reasonably interpreted as the result of the different neuropsychological profile. Taken together, these data highlight the validity of SASG in discriminating aMCI from HC.

To further investigate this issue, we calculated the specificity and sensitivity of SASG and compared them to those of gold standard tests for aMCI detection, the MoCA test and the immediate and delayed recall of the FCSRT. Results of these analyses show that SASG has a very good performance (AUC: 0.879) in discriminating between groups, and these are statistically comparable to immediate and delayed recall of the FCSRT and to MoCA test, as shown with the ROC analysis. This is in agreement with data demonstrating the validity of the FCSRT in detecting memory deficits in subjects at risk of AD, making the use of this test recommended by the International Working Group (IWG) [11]. Finally, SASG results in a significantly higher sensitivity and specificity when compared to the TMT. This result was expected due to lack of involvement of mnemonic abilities in the TMT. Taken together these data demonstrate the validity of SASG in detecting mild neurocognitive deficits involving the memory domain that do not impact the functionality of everyday life such as in the aMCI condition [26].

Finally, the third aim was to test the relationship between the SASG tasks and the hippocampal volumes. Results show that SGSA is comparable to FCSRT and outperforms MoCA in the ability to detect the reduction in the hippocampal volume. This datum is very important since pre-morbid hippocampal volume is predictive of a subsequent clinical progression towards AD and it is thus considered a biomarker [35,36]. In a previous study by Sarazin et al. [60], the FCSRT was proved to be correlated with left hippocampal volume, particularly in the CA1 region in AD patients [60]. In our study we found a significant relationship between SASG performance and right hippocampus volume. Interestingly, this asymmetric relationship can be due to the visual–spatial nature of the task. The right hippocampus has been indeed demonstrated to be involved in memory for locations within an environment, and this corresponds to some of the tasks involved in the SASG evaluation (see [61] for a review).

## 5. Conclusions

In conclusion, this virtual-based tool constitutes an ecological and clinically meaningful task, useful to assess the cognitive profile in subjects with subtle and selective memory complaints such as aMCI subjects. SASG has substantial advantages that make it useful even in a clinical context: it is user friendly, ecological and motivating for the users [10,62]. The integration of technology into cognitive assessment practices provides a new ground for a modern approach to neuropsychology, making it able to digitally collect and combine a higher number of variables in a better evaluation of the behavioral profile. This aspect is of seminal importance and, in this perspective, the SASG represents an ecological tool for the timely detection of the functional impairment of this clinical condition, as recommended in the practical guidelines on MCI of the American Academy of Neurology [28]. Moreover, the significant relationship of SASG performance with the right hippocampal volume demonstrates how results on this task hold the potential to offer a putative digital biomarker able to capture the aMCI condition. The herein presented data are relevant because they show the efficacy of SASG in recognition of patients at risk to develop AD in a pre-clinical stage. Considering the lack of pharmacological treatments for this condition, early detection of subjects at risk is decisive for the implementation of timely and effective rehabilitation interventions as the only opportunity to reduce the risk and the impact of the cognitive decline.

## Figures and Tables

**Table 1:** Group comparison to compare healthy controls (HC) and amnestic mild cognitive impairment (aMCI) groups for demographic variables.

	aMCI	HC	*p*-Value [η2; Observed Power]
*n*	32	107	
Age, yrs (mean ± SD)	76.75 ± 5.31	76.47 ± 3.03	n.s.
Gender (M:F)	17:15	54:53	n.s. #
Education, yrs (mean ± SD)	10.75 ± 3.84	10.95 ± 4.09	n.s.
MMSE	27.65 ± 1.79	28.74 ± 1.27	0.0027
N-Hipp volume	*n* = 30	*n* = 27	
Left n-Hipp (mean ± SD)	0.001710 ± 0.0000	0.002055 ± 0.0000	<0.001 ** [0.22; 0.96]
Right n-Hipp (mean ± SD)	0.001783 ± 0.0000	0.002099 ± 0.0000	<0.001 ** [0.26; 0.99]

*n*: number of subjects; yrs: years; SD: standard deviation; M: male; F: female; MMSE: mini mental state examination; N-Hipp volume: normalized hippocampal volume; # Chi-squared Test; ** ANOVA = age and education as covariates and (η2 partial value; observed power).

**Table 2:** Frequency of use for technological devices in our sample.

	aMCI	HC	*p*-Value
*n*	32	106	
Frequency of computer use			
Infrequent % (*n*)	53.12% (17)	60.38% (64)	n.s. #
Frequent (at least once a week) % (*n*)	46.88% (15)	39.62% (42)	
Frequency of use of a touch screen (last year)			
Never % (*n*)	62.5% (20)	72.64% (77)	n.s. #
Infrequent % (*n*)	18.75% (6)	20.76% (22)	
Frequent (at least once a week) % (*n*)	18.75% (6)	6.60% (7)	

*n*: number of subjects; # Chi-squared test; n.s.: not significant.

**Table 3:** Neuropsychological evaluation. ES = Equivalent Score. ES = 0 pathological score; ES = 1 borderline range.

Test	aMCI	HC	Cut-off (ES = 0)	ES = 1	*p*-Value
*n*	32	107			
MoCA (mean ± SD)	22.26 ± 2.84	26.97 ± 2.35	≤15.50	15.51–18.28	**<0.001** *
FCSRT (median, IQ range)					
IFR adjusted	19.59 (15.79 to 23.80)	27.38 (25.38 to 29.42)	≤19.59	19.60–22.53	**<0.0001** ^§^
ITR	36 (33.5 to 36.00)	36 (36.0 to 36)	<35	--	**0.0005** ^§^
DFR adjusted	6.12 (2.67 to 10.16)	9.67 (8.67 to 10.89)	≤6.31	6.32–7.66	**<0.0001** ^§^
DTR	12 (9.5 to 12.0)	12 (12 to 12)	<11	--	**<0.0001** ^§^
TMT (median, IQ range)					
TMT-A	39 (21.5 to 64.0)	40 (28.0 to 59.0)	>93	93–69	n.s ^§^
TMT-B	91 (55.0 to 182.75)	77.5 (49 to 115)	>282	282–178	n.s. ^§^

*n*: number of subjects; MoCA: Montreal cognitive assessment—adjusted total scores [46]; FCSRT: free and cued selective reminding test [48]; IFR: immediate free recall; ITR: immediate total recall; DFR: delayed free recall; DTR: delayed total recall; TMT: trail making test [49]; in bold = *p*-values surviving Bonferroni’s correction (*p* < 0.007) * One-Way ANOVA; ^§^ Mann–Whitney test.

**Table 4:** Smart aging serious game (SASG) evaluation, expressed in z-scores. Mann–Whitney test. Effect size (d) and power are also included for each statistic.

Test	aMCI (Mean, IQ)	HC (Mean, IQ)	*p*-Value	Effect Size (d)	Power (1-β err prob)
SASG Accuracy Index (AI)					
Task 1	−0.96 (−1.60 to −0.31)	0.12 (−0.31 to 0.55)	**<0.0001**	1.04	0.99
Task 2	0.48 (0.10 to 0.74)	0.23 (−0.28 to 0.68)	0.040	0.33	0.36
Task 3	−0.78 (−0.78 to 0.83)	0.83 (−0.78 to 0.83)	0.026	0.45	0.59
Task 4	−0.99 (−2.39 to 0.15)	0.41 (−0.15 to 0.98)	**<0.0001**	0.94	0.99
Task 5	−1.75 (−2.51 to −0.63)	0.50 (−0.06 to 0.50)	**<0.0001**	1.28	0.99
SASG Time index (TI)					
Task 1	1.02 (0.72 to 1.53)	−0.06 (−0.70 to 0.65)	**<0.0001**	1.26	0.99
Task 2	0.63 (0.28 to 1.40)	−0.01 (−0.57 to 0.50)	**<0.0001**	0.90	0.99
Task 3	1.03 (0.69 to 1.46)	−0.19 (−0.78 to 0.77)	**<0.0001**	1.19	0.99
Task 4	0.45 (−0.20 to 0.69)	0.15 (−0.78 to 0.74)	n.s.	0.34	0.37
Task 5	1.33 (1.28 to 1.38)	−0.30 (−0.90 to 1.19)	**<0.0001**	1.81	0.99
SASG−Total	−8.29 (−12.04 to −4.90)	0.84 (−3.07 to 3.71)	**<0.0001**	1.61	0.99

SASG-total: smart aging serious game total score; in bold = *p*-values surviving Bonferroni correction (*p* < 0.005).

**Table 5:** Statistical comparison between the SASG ROC curve and other tests in terms of sensitivity, specificity, AUC, SE and confidence intervals.

Test	Sensitivity	Specificity	AUC	SE	95% CI	*p*-Value	Criterion Value (J Index)
SASG-Total	84.4	75.5	0.88	0.03	0.81 to 0.92	--	≤−3.28 (0.60)
MoCA (adj)	71.9	90.6	0.89	0.03	0.81 to 0.94	n.s.	≤23.44 (0.62)
FCSRT – DFR (adj)	65.6	97.2	0.76	0.06	0.67 to 0.83	n.s.	≤6.78 (0.63)
FCSRT – IFR (adj)	71.9	91.5	0.85	0.05	0.79 to 0.91	n.s.	≤22.35 (0.63)
TMT-A (adj)	15.6	95.3	0.52	0.06	0.43 to 0.60	<0.0001	≤14 (0.11)
TMT-B (adj)	40.63	84.91	0.61	0.06	0.53 to 0.70	0.0001	>130 (0.25)

Results have been considered as statistically significant when surviving Bonferroni corrected threshold, *p* < 0.01. *p*-value = comparison with SASG-Total. AOC: area under the curve; SE: standard error; CI: confidence interval; J: Youden J statistic; SASG-total: smart aging serious game total score; MoCA: Montreal cognitive assessment; FCSRT: free and cued selective reminding test; DFR: delayed free recall; IFR: immediate free recall; TMT: trail making test; adj: adjusted.

**Table 6:** Partial correlations between test and normalized hippocampal volume. Age, gender and education have been included as covariates of no interest. Results have been considered as statistically significant when surviving Bonferroni-corrected threshold (*p* < 0.008).

		Left n-Hipp	Right n-Hipp
*n*		30	30
**SASG-Total**			
	*Corr*	0.28	0.50
	*p-value*	n.s.	0.0076
**MoCA**			
	*Corr*	−0.08	0.06
	*p-value*	n.s.	n.s.
**FCSRT-IFR/DFR**			
	*Corr* *p-value*	0.30/0.4673 n.s./0.0140	0.39/0.48 0.046/0.011
**TMT-A/B**			
	*Corr* *p-value*	0.29/0.24 n.s./n.s.	0.15/0.08 n.s./n.s.

n-Hipp: normalized hippocampal volume; SASG-total: smart aging serious game total score; Corr: correlation coefficient r; MoCA: Montreal cognitive assessment; FCSRT: free and cued selective reminding test; DFR: delayed free recall; IFR: immediate free recall; TMT: trail making test; n.s.: not significant.
